# Athletic Races Represent Complex Systems, and Pacing Behavior Should Be Viewed as an Emergent Phenomenon

**DOI:** 10.3389/fphys.2018.01432

**Published:** 2018-10-05

**Authors:** Andrew Renfree, Arturo Casado

**Affiliations:** ^1^Institute of Sport & Exercise Science, University of Worcester, Worcester, United Kingdom; ^2^Department of Physical Education, Isabel I University, Burgos, Spain

**Keywords:** decision-making, pacing, endurance, emergence, competitions

## Abstract

Pacing is the manner in which effort is distributed over the duration of an exercise bout, and is an important determinant of the extent to which individual potential is realized during athletic races. Observed pacing behaviors are thought to result from complex decision-making processes, and several models have been proposed that may explain the manner in which these decisions are made. In this article we argue that examination of individual factors implicated in the regulation of pacing is unlikely to allow full understanding of the events leading to pacing and performance. Rather than utilizing such a reductionist approach, it is suggested that athletic races be viewed as complex systems, and that pacing behavior is an emergent phenomenon that cannot be fully understood through study of components of the system in isolation. We describe and discuss known and potential interactions between determinants of pacing during races, and conclude with a call for the development of novel research methodologies that may further understanding of the manner in which observed behaviors emerge.

## Introduction

Participation in athletic racing events requires regulation of exercise intensity, and the way in which effort is distributed throughout an exercise task is termed pacing (Foster et al., 1994). Pacing is achieved through continual decision-making (Renfree et al., 2014; Smits et al., 2014), as athletes select from all exercise intensities between rest and the maximal they are capable of generating at that moment. Decisions regarding strategic approach are made before exercise, whereas tactical decisions during the event are in response to changes in physiological status (St Clair Gibson et al., 2006). Even short sprint events require an optimal distribution of effort, typically characterized by an ‘all out’ strategy, if potential is to be realized (Abbiss and Laursen, 2008). In addition to ‘internal’ factors, performance relative to goals and other competitors also influence pacing decisions. A race comprising numerous participants may therefore be considered a complex system composed of heterogenous individuals who interact (Balague et al., 2013). Individuals are adaptive, and continually change behaviors in response to emerging constraints as they continue to strive to achieve goals. These continuously changing behaviors further increase complexity, and make predictions of the behavior of individuals, and the system as a whole, difficult. This means that the determinants of pacing behaviors may differ greatly between individual time-trial type events and mass start ‘race’ events, even if there are similarities in terms of event duration or metabolic demands. We may speculate that in the former, psychobiological variables such as Rating of Perceived Exertion (RPE) (Tucker, 2009) or affect (Baron et al., 2011) are the dominant variables, whereas performance relative to competitors may be more important in the latter (Smits et al., 2014). Furthermore, the experience level of athletes may well influence pacing behaviors. Foster et al. (1994) reported that athletes ‘learned something’ by attempting pacing strategies they had not previously utilized. Athletes with greater competitive experience will therefore have been exposed to a wider range of competitive scenarios. In effect then, it seems that pacing behaviors emerge from the interplay of internal physiological factors specific to individual competitors, and external factors relating to the characteristics of the competitive environment. The nature of the interactions between these variables is explored further in the next section of this paper.

## Complex Systems and Emergent Phenomena

All biological systems are complex, and complexity is a characteristic of any system which displays properties that are unpredictable based on knowledge of its components. These unpredictable characteristics which arise from the interactions between the components of the system are termed the ‘emergent’ properties of the system, and it is the presence of these emergent properties that makes a biological system ‘complex’ (Wilkins, 2002). A characteristic of complex biological systems is that self-organization occurs in a way that cannot be explained through examination of constituent components in isolation (Macklem, 2008), meaning that a reductionist approach is unable to explain characteristics of the system as a whole (Mazzocchi, 2008). This suggests even complete knowledge of the components cannot allow prediction of behavior, meaning characteristics displayed by the system are emergent (Mayr, 1982). Emergent phenomena occur at various levels within biology, including the molecular, cellular, organism, ecosystem, and societal (De Haan, 2006). This implies some hierarchical level of organization, as attempts to explain behaviors at one level rely on different mechanisms to explain those at a different level. As all biological systems are complex (Wilkins, 2002), we suggest all behaviors in the sporting arena should be considered emergent phenomena. However, for the remainder of this manuscript we will examine the phenomenon of pacing in athletic races and the nature of the interactions that may lead to observed behaviors.

Existing models of endurance performance do suggest hierarchical organization. In considering the physiological determinants of endurance performance, Joyner and Coyle (2008) present a framework describing the factors that interact to determine performance velocity. Performance power is determined by aerobic and anaerobic abilities along with mechanical efficiency. These physiological qualities in turn result from morphological characteristics including muscle capillary density, cardiac output, and muscle fiber composition. In this purely physiological model, the interactions between the various subsystems would suggest performance prediction based on knowledge of individual components is unlikely (Mayr, 1982). In reality the determinants of endurance performance are more complex. Indeed, this model presents physiological determinants of endurance *potential* rather than performance *per se*. Although an athlete may display physiological qualities associated with elite performance, there is no guarantee this will be achieved. Noakes (2008) made this point when suggesting an over-emphasis on maximal oxygen consumption has resulted in a brainless model of performance that does not reflect the reality of competitions where athletes must regulate exercise intensity, emphasizing that existing purely physiological models used to explain exercise performance are incomplete. Although the regulation of exercise performance is not fully understood, a complex systems model was developed by Lambert et al. (2005) which proposes control processes are modulated in a dynamic and non-linear manner. A key feature of this model is that there is no single regulatory component. Rather, multiple levels of regulation together achieve homeostatic control. St Clair Gibson et al. (2018) further developed this model by suggesting dynamic control is generated through not only physiological homeostatic drives, but also psychological drives such as motivation. These physiological and psychological drives are in competition, and result in dynamic oscillations in the activity of all systems.

Although pacing behaviors in athletic competition result from conscious decision-making processes (Renfree et al., 2014; Smits et al., 2014), there are competing theories as to how this operates. Ulmer (1996) initially proposed the process of teleoanticipation whereby a mathematical algorithm is created in the brain before exercise. In this model, motor drive is varied based on afferent feedback, and metabolic control is achieved through reference to the RPE. A number of different regulatory models have subsequently been developed, but all are in agreement that the RPE plays an important role (Tucker, 2009; Marcora, 2010; de Koning et al., 2011). Regardless of the precise mechanisms through which RPE is generated, and subsequently informs decision-making, it is evident that RPE is liable to disruption via non-physiological factors including accuracy of distance feedback (Baden et al., 2004), and the presence of intermittent crowd support (Noakes, 1992). These observations suggest that behavior therefore emerges from complex interactions between athletes physiological and psychological state, and the characteristics of the environment in which they are competing.

In addition to RPE, emotion informs pacing decision-making. Baron et al. (2011) identified a role for affect, a representation of feelings experienced within a situation (Watson, 2000), in regulation of exercise, and suggested a more positive state leads to increased motivation. A key determinant of the affective response to exercise is interpretation of the current situation (Watson, 2000), suggesting performance relative to competitors is a determinant of motivation to continue. Indeed, Jones et al. (2016) found competition against an avatar deceptively set to a speed 2% faster than participants believed, resulted in more negative affect even though performance was improved. Venhorst et al. (2018) also found that falling behind an opponent during a 70 km time trial resulted in deterioration in affective valence and increased endocrinological stress response. Although the relationship between performance time, race position, affective response, pacing decisions, and physiological consequences is complex, it seems behaviors ultimately emerge from this series of interactions in a manner that cannot be predicted through understanding of individual factors in isolation.

Other work on the determinants of pacing has emphasized the importance of athlete interactions. Studies in the field (Hanley, 2013, 2014, 2015, 2016; Renfree and St Clair Gibson, 2013; Esteve-Lanao et al., 2014; Konings et al., 2016a; Noorrbergen et al., 2016; Renfree et al., 2016) and laboratory (Konings et al., 2016b) demonstrated pacing decisions are influenced by behavior of other competitors, possibly due to a tendency to engage in collective behavior (Renfree et al., 2015). Rather than resulting from purely ‘internal’ processes, it therefore seems pacing decisions are also influenced by the environment in which they are made, as is the case within team sports. Smits et al. (2014) suggest that rather than being reliant on information stored in the brain, athletes utilize information that becomes available in a specific situation to inform decisions. Behavioral options continuously change due to fluctuations in individual capabilities and the features of the environment. Participants utilize affordances, which are utilized to identify behavioral actions, and action capabilities which refer to the ability to act on this information. These action capabilities are determined by physiological and psychological state, which are themselves liable to change due to progressive physiological disruption and reassessment of current performance.

To summarize to this point, regulation of pacing during athletic competition relies on continuous decision-making. This process is informed by internal physiological conditions, psychological state, and behavior of other competitors. Due to the complexity of biological processes internal to the individual, and within the broader competitive environment, pacing behaviors are unlikely to be predictable based on knowledge of individual contributors to the regulatory process. It is therefore suggested that pacing behavior is emergent and cannot be fully explained through study of its constituent components.

## Emergent Human Behavior in Group Environments

One human environment in which emergent behavior has been studied is within large crowds. As in other models, a hierarchical organization exists, consisting of three different levels: the individual, interactions between individuals, and the group (Pan et al., 2007). Individual behavior results from internal decision-making processes, while interactions are influenced by social structures, and the group is influenced by factors such as crowd density, environmental constraints, and peer imposed mental stresses. Although this model refers to a non-sport situation, it is possible to suggest similar principles apply during athletic competition. Internal processes informing decision-making have already been identified, and include knowledge of the endpoint, interpretation of afferent feedback, RPE, and emotion. With regards to interactions between individuals, it is becoming clear these do influence individual decision-making. In the laboratory, Konings et al. (2016b) found competition against a virtual opponent during cycle time trials resulted in higher starting speeds. Subsequent work by Konings et al. (2018) demonstrated competition resulted in not only improved performance, but also a greater decline in muscle force, suggesting athlete interactions influence the degree to which physiological status is disrupted. With regards to group level factors, several studies have identified that endurance competitors make remarkably similar pacing decisions, at least in the early stages of an event (Hanley, 2013, 2014, 2015, 2016; Renfree and St Clair Gibson, 2013; Esteve-Lanao et al., 2014; Renfree et al., 2016).

Previous work has already suggested behaviors in sporting environments are emergent, although this has largely been within team sports. Duarte et al. (2012) likened sports teams to ‘superorganisms’ whereby overall behavior results from collective processes governing individual interactions. Indeed, they go on to suggest sports team analysis would be best served by adopting models explaining how social collective behaviors emerge from interactions between individuals. Actions by one individual generates information that can be utilized by other individuals or groups in informing subsequent actions. Importantly, individuals possess differing characteristics, meaning they display their own idiosyncratic behaviors. Individuals differ in terms of a range of characteristics including physiological capabilities, psychological traits and states, and technical abilities, thereby suggesting decisions regarding behavior emerge from not only interaction rules, but also the ability of individuals to continue to act upon information.

Some evidence for pacing behaviors being emergent is provided by Trenchard (2015) who studied collective behaviors in bicycle pelotons. At low speeds, cyclists share energetically costly leading positions and positional changes are frequent. As speeds increase, weaker athletes can maintain contact with stronger riders only by adopting following positions. As speeds increase further, weaker cyclists cannot maintain contact despite drafting, and the peloton breaks up. The precise speeds at which these events occur depends on differences in sustainable power outputs between riders and the drafting benefit. This benefit depends on speed, and increases as speed becomes higher. If we consider the situation when the peloton begins a climb, speed decreases even though power output stays the same or increases. Due to reduced speed the drafting benefit is also reduced, meaning riders who were able to maintain contact on the flat now cannot. The shape and speed of the peloton therefore emerges from interactions between individual athletes, speeds, wind direction, and course topography. Due to the role of perception of individual performance relative to others in determining psychological responses and performance (Jones et al., 2016; Konings et al., 2018; Venhorst et al., 2018), it also seems that peloton level dynamics may affect subsequent individual decision-making. This example demonstrates that observed behavior is emergent because it results from the interactions between physiological, psychological, and purely physical factors.

The example above relates to cycling, where drafting dominates behavior due to the energetic savings available. In running however, the energetic savings are smaller and may have less influence on behaviors. In a study that used computer modeling to simulate the effect of three different drafting quantities (i) no drafting benefit, (ii) realistic (8%) runner drafting, and (iii) unrealistic (35%) ‘cycle’ drafting, Trenchard et al. (2017) found drafting had minimal impact on three measures of collective behavior. A realistic 8% benefit resulted in significant, but small, increases in speed in a simulated 10000 m running race, but no effect on Runner Conversion Ratio (the drafting benefit experienced by the follower in a pair) or distance between the leading and last placed runner. As analyses of actual competitions suggest competitors in running events do display forms of collective behaviors such as similar starting speeds (Renfree and St Clair Gibson, 2013; Esteve-Lanao et al., 2014; Hanley, 2014) and pack formation (Hanley, 2015), it may be that these behaviors emergence is based primarily on other, presumably cognitive, factors. Given that these modeling studies have demonstrated that the influence of drafting on pacing differs between different sports, it may be the case that the primary drivers of emergent pacing behavior differs between sports, with drafting being dominant in the ‘high speed’ events, and cognitive factors being more important in shorter events.

A key feature of groups of organisms is that differences in individual decision rules have implications for social dynamics. Kenrick et al. (2003) suggest that even if a small number of individuals within a population have a low threshold before displaying a behavior, (e.g., individuals with a ‘short fuse’ have a low threshold for displaying anger) this can influence behaviors of others. Changes in decision rules followed by some of a population may change decisions made by others, even if their own underlying decision rules are unchanged. In an endurance competition, one can imagine such a scenario. For example, assessment of risk influences starting speeds in novice and experienced individuals (Micklewright et al., 2015). At the start of a race, a small number of individuals with low perceptions of risk may start quickly. This decision may then result in others making the same decision. Even if they are risk adverse, it may be considered more risky to not follow these fast starting rivals. In this situation, the behavior of the group as a whole would have emerged from individual decisions and the influence of social interactions, and would not have been predictable based on just (for example) participants physiological abilities.

In other sports, features of the environment in which the activity is performed also influence the behaviors displayed. In swimming, since the aquatic environment offers a high degree of resistance, the propulsion provided by each stroke emerges from interactions between the fluid and the swimmers actions (Guignard et al., 2017). The relationship between the environment and propulsive forces generated during each stroke is further complicated during open water events, where water can be turbulent but beneficial drafting effects may be obtained. These drafting benefits are influenced by position relative to a leading swimmer (Brisswalter and Hausswirth, 2008). During rowing, the pacing of the boat emerges from interactions with the water and the work performed by individual crew members. A case study of individual contributions to overall boat pacing over 2 and 5 km found that, although whole boat pacing was similar to that typically described for individual ergometry trials, there was considerable variation between individuals within a crew of four (Renfree et al., 2012). Further evidence for the influence of behavior of other crew members on individual stroke action during sculling is provided by Feigean et al. (2017) who studied changes in stroke characteristics in a newly formed coxless pair over a 6 week period. Whilst crew members initially displayed differences in stroke variability, over time the more variable of the pair became more consistent whilst the more consistent of the pair became less so.

## Research Implications

Because of the complex determinants of pacing behavior, performing research to understand these processes is fraught with methodological difficulties. Micklewright et al. (2017) identified limitations of much existing research which involves no manipulation of variables and describes measures of pacing, such as speed or power, whereby data is typically averaged over numerous intermediate segments. The relative size of these segments varies from study to study, meaning a potential loss of ability to detect subtle changes in behavior. Even when segment sizes are relatively small, they may be insufficient to identify outcomes of continual decision-making, especially as even laboratory time trials are characterized by oscillatory fluctuations in power output (Tucker et al., 2006).

Although analyses of pacing during competition describe changes in speed of various sub-groups of participants, (e.g., Hanley, 2013, 2014; Renfree and St Clair Gibson, 2013; Esteve-Lanao et al., 2014; Renfree et al., 2016) many do not describe positional changes. Given the influence of other competitors in informing pacing decisions, this is a potentially important omission. In the previously described peloton model of Trenchard (2015), at low speeds cyclists changed positions frequently, and shared leading positions. Therefore, speed alone provides insufficient information to understand the regulation of exercise intensity by individuals. Even if peloton speed remained constant on a flat road, power output of individuals would fluctuate continually as they swapped positions. Although energetic benefits of drafting during running are smaller, (∼4% reduction in VO_2_ at 6 m.s^−1^) (Davies, 1980; Kyle, 1988) some variation in exercise intensity required to maintain average speed of a group would be expected as athletes change positions. It must also be acknowledged that in mass start competitions the goal is often to achieve a high a finishing position, regardless of completion time. In such situations, positional changes may provide more meaningful information regarding pacing decisions.

Duarte et al. (2012) argue that because of these relationships between individuals, analysis of team sports would benefit from use of biological models explaining how collective social behaviors emerge from repeated interactions between group members. A number of models are proposed on the premise that teams can be conceptualized as ‘superorganisms,’ and team behaviors emerge from locally generated sources of information relating to the position of other players and motions of direction (Duarte et al., 2012). However, endurance sports differ from team sports in a number of respects meaning existing models of performance analysis may be unsuitable for understanding pacing behavior. Whilst team sport players can move in any direction (subject to rules of specific sport), endurance athletes all move in the same direction, whether that be around a track, down a pool, or around a marked course. Furthermore, to achieve their best possible performance, endurance athletes must fully realize physiological potential and incur high levels of fatigue. Although team sport players undoubtedly also experience fatigue, high levels of physiological disruption are not necessarily a pre-requisite for achievement of performance goals. This means any modeling of performance in endurance competitions must also consider individual characteristics. Although physiologically less able athletes may be able to engage with others early in a race, over time accumulated physiological disruption would reduce the number of behavioral options available. We consider that this represents a limitation of some largely ‘physical’ modeling studies (e.g., Trenchard, 2015; Trenchard et al., 2017) which utilize rather crude measures of physiological capacity, and incorporate no psychological variables.

## Future Perspectives

Numerous factors influence pacing behavior in endurance competitions. These include physiological and psychological characteristics, and interactions between individuals and the environment. Although study of such issues in isolation helps us understand their contributions to pacing, this may lead to oversimplification of a complex phenomenon. We propose endurance competitions be viewed as complex systems, and pacing behavior as an emergent phenomenon resulting from continual decision-making performed in the context of momentary status, behavior of other competitors, and environmental conditions. Understanding of the interactions between these variables is necessary to better explain the determinants of observed pacing behaviors. The challenge for researchers in the field is to develop novel methodological approaches that take into account the emergent nature of this phenomenon.

## Author Contributions

AR devised and drafted the study and critically revised the intellectual content. AC revised the study critically for intellectual content and approved the final version to be published.

## Conflict of Interest Statement

The authors declare that the research was conducted in the absence of any commercial or financial relationships that could be construed as a potential conflict of interest.
